# Job Demands and Job Resources of Academics in Higher Education

**DOI:** 10.3389/fpsyg.2021.631171

**Published:** 2021-06-23

**Authors:** Mineshree Naidoo-Chetty, Marieta du Plessis

**Affiliations:** Department of Industrial Psychology, University of the Western Cape, Cape Town, South Africa

**Keywords:** job demands, job resources, qualitative, academics, higher education

## Abstract

Too many job demands and not enough job resources can negatively influence the well-being of employees. Currently, limited information exists surrounding the job demands and resources as experienced by academic employees in the higher education sector. Therefore, the aim of this study was to identify the job demands and job resources experienced by academic employees using qualitative methods. Semi-structured interviews were conducted with 23 academic employees, using an Interpretative Phenomenological Approach. Thematic analysis, specifically template analysis was used to categorize the themes. Job demands were divided into three categories: quantitative (publication pressure, overburdened with the load, and competing time demands), qualitative (work/home balance, complexity of student support, organizational politics, and lack of mental health support) and organizational demands (using technology-mediated learning and lack of structural resources). Job resources were organized into two categories: organizational (social support) and personal resources (autonomy, meaningful work, and personal support). Participant experiences are highlighted to provide a better understanding of the job demands and job resources encountered. The framework of job demands and job resources gleaned from the study could be used for further research to manage and monitor motivational processes for academic staff, and to reduce strain due to high job demands.

## Introduction

South African higher education has undergone a number of transformations since the advent of democracy. Changes in the higher education system and its institutions has been one of the top priorities of the South African government in the post-apartheid era. As such, the Higher Education Sector was identified as a sector in need of review (National Development Plan 2030, 2012). Goals such as increasing and broadening participation, providing equity of access and fair chances of success to all, and decolonisation, as part of the post-1994 transformational efforts (Badat, 2010; Dhanpat et al., 2019), has led to dissatisfaction, on a number of levels, experienced by academics (Pienaar and Bester, 2009). One of the reasons for this dissatisfaction is that although changes and transformations driven by policy might convey the perception that overall progression and betterment is taking place (Van Niekerk and Geertsema, 2009), in actuality these changes have put more pressure on Higher Education Institution (HEI) staff, specifically, academic employees (Dhanpat et al., 2019).

The demands that have been placed on academics, including expanding student numbers resulting in increased academic workload, seem to be a prevailing theme in academic career literature (Theron et al., 2014). These changes are likely to influence employees' work as they experience specific career dilemmas, namely, increased levels of job dissatisfaction (Phillips and Connell, 2003), intention to leave, breach of psychological contracts, break in employee–employer relationships, decline in commitment and job security, and increased workload (Theron and Dodd, 2011).

Job resources are those elements that assist when job demands become excessive (Demerouti et al., 2001). The Job Demands-Resources (JD-R) model puts forward that resources play an integral part in the prevention health-impairment process, and places employee well-being at the focal point. Thus, the resources of academics that assist them in successfully coping with their job demands, is a motivational process that leads to higher levels of work well-being (Byrne and MacDonagh, 2017).

### Job Demands and Resources in Context

For the purpose of this research, the job demands–resources (JD-R) model (Demerouti et al., 2001) will be used as the theoretical framework. Job demands are the physical, psychological, social, or organizational features of a job that need continued cognitive and emotional energy or abilities and are linked with physiological and psychological costs (Demerouti and Bakker, 2011; Bakker et al., 2014). Job resources on the other hand denote the physical, psychological, social, or organizational aspects central to work performance (Rothmann and Jordaan, 2006). These aspects relate to employee experiences of job satisfaction, autonomy, purpose, engagement, and meaningful work, and job performance (Janse van Rensburg et al., 2018).

Job demands can be divided into three categories, namely, quantitative demands, qualitative demands and organizational demands whilst resources can be organized into two categories, namely, organizational resources and personal resources (Schaufeli, 2017). Quantitative demands refer to elements such as the number of tasks and the speed at which it can be accomplished. When quantitative work demands are elevated, work tasks will necessitate more time than what was planned for (Van Veldhoven, 2014). Qualitative demands focuses on the type of skills and/or effort required to complete work tasks. For example, cognitive, emotional or physical skills and/or effort. It refers to the level of difficulty or complexity that is needed to carry out the job (Bowling and Kirkendall, 2012). In addition, organizational demands negatively impact an individual in terms of their work outputs (Bakker et al., 2004), and can be described as those elements that are brought about by aspects in the work environment.

Resources are categorized according to organizational and personal resources. Organizational resources are mostly retrieved from external sources such as a supervisors or co-workers. These resources may include feedback, rewards, job control, participation, job security, and supervisor support (Demerouti et al., 2001). Personal resources focus on the views and judgement of the individual (Hoy, 2004; Barrick et al., 2013), i.e., meaningfulness of work, autonomy, self-efficacy, optimism and organizational-based self-esteem (Xanthopoulou et al., 2009).

Whilst other job stress models, such as the demand-control model (Karasek, 1979) and the effort-reward imbalance model (Siegrist, 1996) also explain the predictive value of job characteristics in employee well-being, the limited set of predictor variables in these models may not be relevant to all occupations (Bakker and Demerouti, 2007). Therefore, the JD-R model was used to guide the exploration of what demands and resources exist in the academic environment.

### Demands and Resources in the South African University Setting

Universities in sub-Saharan Africa continue to operate under conditions that are under-resourced, with academic staff experiencing high workloads that negatively affect their well-being and performance (Higher Education South Africa, 2014). Several studies [see for example Rothmann and Jordaan (2006), Barkhuizen and Rothmann (2008), Pienaar and Bester (2009), Bezuidenhout and Cilliers (2010), Barkhuizen et al. (2014a,b), and Van Tonder and Fourie (2015)] reviewed aspects of job demands and resources and their implications in a Southern African higher education setting from a quantitative perspective.

This can be further supplemented by research carried out in other parts of Africa. Reports have indicated that teaching at a university level is a high stress occupation. In Ghana, the main stressor for lecturers was the augmented intake of students with no consideration for expansion of university facilities (Atindanbila, 2011). As a result, lecturer-student ratio results in work overload. Teaching load and professional distress were also rated as the second and third highest sources of tension experienced. Furthermore, academic staff in Egypt indicated that demands such as poor working conditions, limited career development, increased levels of work overload and insufficient resources contributed to difficulties experienced in the academic environment (El-Sayed et al., 2014).

South African academics additionally reported pressure to produce more research outputs, bigger classes and postgraduate supervision loads as sources of strain. Coping with a high volume workload was further exacerbated by the experience of unclear roles and responsibilities (Bezuidenhout and Cilliers, 2010).

Accordingly, it appears that the job demands of academics have escalated, whilst the levels of support and other resources have declined (Barkhuizen et al., 2014a). According to literature, job resources can lead to an engaged workforce, which is a key value proposition for Higher Education institutions that aim to retain talented staff (Barkhuizen et al., 2014a). As job demands for academics are consistently negatively related to work engagement and well-being (Naidoo-Chetty and Du Plessis, 2021), the need for further studies in this area is highlighted. Pon and Lichy (2015) note that there is limited research being done based on the experiences of academic employees in the academic field on job demands and resources, nationally and internationally.

### Rationale of the Study and Problem Statement

From investigating the literature on job demands and resources, it is clear that most studies have engaged in quantitative methods. While these studies have made a noteworthy impact, quantitative methods have some restrictions as it is less flexible and exploratory in nature (Queirós et al., 2017). Empirical results were also obtained based on the researchers' theorizing on the job demands and resources of academics, thus presenting a perceived objective reality of academic demands and resources. One aim of this study was to identify the job demands and resources as experienced by academics by using qualitative methods. This allowed us to capture the subjective feelings of the academic working environment and what is experienced as demands and resources.

A deeper and richer understanding of academic employees' experiences of job demands and job resources, will permit academics to better manage their career demands and resources. Furthermore, it can help organizations to assist employees. When an organization fails to make essential job resources available, there is the possibility that employees will withdraw and disengage, which may lead to burnout (Takawira et al., 2014). However, even though there is clear evidence indicating that establishing engaging work environments is vital, the current climate at higher education institutions is rather somber (Geoffrey and January-Enkali, 2019). These types of problems have been reported as far back as 2006 by Rothmann and Jordaan in the South African higher education system. Aspects such as imbalances and misrepresentation of the system, poorly equipped students, and diminishing government funding, continue to be problems in need of addressing.

Therefore, the current study is guided by the following research question: How do academics make sense of their experiences of job demands and resources in higher education?

## Materials and Methods

### Participants and Setting

All participants were from a selected public University in the Western Cape, South Africa. An invitation e-mail to participate in the interviews was sent to all academic staff by the respective university. When this method produced a low response rate, snowball sampling was utilized to improve the responses. In total, 23 semi-structured interviews were conducted with the majority of the participants being female. In total, there were 18 females and five males. The number of interviews represented an adequate sample size as it allowed analysis to saturation. As per the phenomenological research tradition, the number of the participants can be between two and 25 (Creswell, 2012). Of the interviewees, three were Professors, five were Senior Lecturers, and 15 were Lecturers. Within the research university, academics at all levels are involved with teaching, research, community engagement and supervision of postgraduate students to different degrees. Lecturers (typically early career scholars who do not hold a PhD) carry a greater burden of undergraduate teaching, whilst Professors (established researchers) have more postgraduate supervision responsibility.

The research institution is classified as a historically disadvantaged institution, and has been the vanguard of South Africa's historic change, with a distinctive academic role in helping to build an equitable and dynamic nation. Coming from a history of creative struggle against oppression, discrimination and disadvantage, the university played an important role in helping to build equitable access to education. In 1982, the university formally rejected the apartheid ideology and adopted a declaration of non-racialism. The University also formalized an “open” admissions policy which provided access to a number of African students, as well as an expansion to the curriculum taught to equip students to be successful and employable in the workplace. Now, the university (depending on the ranking being used) ranks within the first tier of Universities in South Africa based on research output.

### Data Collection

To understand and describe the job demands and job resources academics experience, the researcher conducted semi-structured online video interviews with each participant. Online video interviews were considered the best method to use at the specific point in time due to the COVID-19 pandemic and the subsequent requirement to socially distance. Moreover, the semi-structured interview is the exemplary method for Interpretive Phenomenological Approach (IPA) as it facilitates rapport and allows the interview to go into novel areas (Smith et al., 2009). Interviews also included sub-questions (probing questions) to further probe for more detailed or “richer” responses from the participants, where it was required. Some of the questions that participants were asked in the interview were the following— “What resources in your work environment play a role in making what you do a success?;” “What are the work challenges that excite you;” “what work challenges makes it difficult for you to do and be your best?;” “What, in your view, are the types of work pressures that you experience in your work?” Furthermore, the interviewer asked participants to assign a score of 1–5 to provide a rating of the intensity of the demand that impacted the academic the most on a day to day basis.

At the beginning of each interview, the interviewer (the first author) built rapport by disclosing that she herself was an academic employee and had her own set of experiences. Thus, the interviewer ensured that she was transparent and reflexive in her thinking (Polit and Beck, 2014) to ensure that her own perceptions did not impact the process by which the data was collected, analyzed and presented. Furthermore, the interviewer explained to participants that the purpose of the interview was to gain insight into the job demands and resources they experienced in their daily working lives. As the interviewer was not from the same department as participants, and did not hold a position of influence in relation to the participants (such as head of department, senior management, human resource professional, etc.), it is unlikely that her employment in the same institution would have discouraged participation and open sharing.

### Data Analysis

A phenomenological approach was used in order to obtain an understanding of the phenomenon of job demands and resources. Specifically, the interpretive phenomenological analysis (IPA, Smith, 1996) was used in conjunction with Template analysis to gain insight into how academics made sense of their experiences of demands and resources related to their job roles as academic employees within the Higher Education Sector.

The steps for analyzing IPA data as recommended by Smith and Osborn (2008) was followed. Firstly, the transcript was read and reread closely, with the left-hand margin being used to annotate what was interesting or significant about the participant's comments. When the entire transcript was annotated, the researcher returned to the beginning of the transcript and documented emerging theme titles. Here the initial notes were transformed into concise phrases, which aimed to capture the essential quality of what was found in the text. The themes moved the response to a slightly higher level of abstraction and invoked more psychological terminology. This transformation of initial notes into themes was continued through the transcripts with the same theme title used when similar themes emerged.

In terms of template analysis, which is a form of thematic analysis, themes were categorized and identified using the IPA method and thus constructed into a template. The template was divided into quantitative, qualitative and organizational demands. Furthermore, resources were divided into personal and organizational resources.

As a further step to the analysis, Participative Ranking Methodology (PRM) was used to determine the most challenging job demands. This is a “mixed methods” approach to data collection, where a group of experienced participants are directed toward generating responses to a detailed question or set of questions. The reason it is considered a “mixed methods” approach is that it draws on both quantitative and qualitative methodologies which assists in producing rich, contextualized data that can be calculated, ranked, and paralleled across or within groups (Ager et al., 2010). Thus, by using data obtained in the qualitative phase, certain themes became prevalent that were relevant to the academic environment. To calculate the average rank for an issue mentioned by academics we added up the ranking number from the group and divided by the number of participants (Ager et al., 2010).

### Rigor

Credibility was achieved through the involvement of two researchers who analyzed one written interview independently. Furthermore a codebook was developed together by both researchers using verbatim quotes to provide the study participants a voice along with the researchers' data interpretations, respectively (Polit and Beck, 2010; Halcomb et al., 2013). In case of disagreement on the themes, there was an additional discussion to ensure that both researchers were on the same page. Self-reflection was an important consideration for dependability. Researchers were stimulated to put their own ideas on paper before starting the interviews and the analyses. This created for the researcher a constant awareness about their own background and perspective.

### Ethical Considerations

Ethical approval was obtained from the Human and Social Sciences Research Ethics Committee at the Researchers' Institution. Verbal and written consent was obtained from all participants prior to interviews and after verbal and written information about the research goals and methods was provided. Participants were assured that their identity would not be disclosed under any condition. They were free to withdraw from the research process at any time and without any explanation.

## Results

The aim of the research was to identify the job demands and job resources experienced by academics in higher education. As mentioned earlier, the responses of the participants were categorized based on job demands (quantitative, qualitative and organizational) and job resources (organizational and personal).

### Quantitative Demands

Quantitative demands refer to elements such as the number of tasks and the speed at which it can be accomplished. Therefore, themes in this category carry the notion of volume of work or hours needed to accomplish such work, coupled with the available time within which such tasks should be completed. Three themes were identified as quantitative demands.

#### Publication Pressure

The first theme that was identified as a quantitative demands was *publication pressure*. In the participative ranking of job demands, publication pressure was ranked as the most prominent. The responses were mostly related to research outputs. Individuals such as P5, P22, and P23 mentioned feeling stressed and tired from the constant need to produce publication*s*. For example:

“*Teaching does take up the predominant amount of time, which puts pressure on my research. Publishing an article is a long and strenuous process*.” (P5, female, senior lecturer, Faculty of Economic and Management Sciences).

“*Time for research is what remains after teaching and administrative requirements are met. Yet, I feel like the institution puts pressure on me to publish*.” (P22, female, lecturer, Faculty of Arts and Humanities).

“*I have to stress over publishing, in order to get promoted. I have to publish and be running around and going to conferences.”* (P6, female, associate professor, Faculty of Natural Sciences).

In support of P5 and 22's comment was the notion that due to additional time spent on teaching and with the new way of technology-based teaching, academics have gotten to the point where time for research appeared to be that remaining after teaching and administrative requirements had been met (Houston et al., 2006, 25). In fact, most of the academics indicated that “*they did not have a lot of time to focus on their research*” (e.g., P12, P17, P18, and P20). This has led to P6 feeling anxious and worried about not being promoted due to a lack of publications.

Other obstacles to overcome were related to research funding. The competitive nature in obtaining funding caused a lot of anxiety and stress, yet, the university still expects outputs. Participants indicate:

“*You have to apply for funding, you have to apply for leave, you have to prove where you're going, how long you're going, what your outputs are going to be, and then sometimes you even go through this whole admin process and get rejected*” (P5, female, snr lecturer, Faculty of Economic and Management Sciences).

“*Some years you would get, some years we wouldn't get. It all depends on how the competition is at a certain year and how the economy is doing.”* (P6, female, associate professor, Faculty of Natural Sciences)

In South Africa, academics are increasingly pressurized to publish in journals accredited and incentivised by the Department of Higher Education and Training (DHET) in order to be recognized and rewarded for their work (Department of Education, 2015). It can also be noted that Deans and Deputy Vice Chancellors are setting impractical performance management metrics, which include publishing papers in certain journals (Guthrie et al., 2019). Furthermore, these targets are being set in an environment where there is a significant increase in teaching and administration loads (Guthrie et al., 2019).

Kubátová (2019) has noted that publishing forms an integral part of academic work worldwide. A reason for this is that Academic institutions are ranked according to the level of publication in high-impact journals. Research grants are thus awarded to institutions based on their publications. This has also put academics under pressure to produce increasingly higher numbers of research publications as well as research grants, with a particular focus on volume rather than quality (Callaghan, 2016).

#### Overburdened With the Load

An overwhelming number of participants mentioned being *overburdened with the load* that forms part of their work. Through the participative ranking method, this job demand was placed as second. For instance, participants mentioned:

“*[C]lass numbers are very big*” (P1, female, lecturer, Faculty of Community and Health Sciences)

“*You want to help the students and you want to be able to get to those who are falling behind, but because classes are so big, it's often actually difficult to make a difference.”* (P23, female, senior lecturer, Faculty of Law).

“*[T]here is always additional work and the role is not clear at times*” (P13, female, lecturer, Faculty of Dentistry)

“*I have hundreds of students. If I spent 10 minutes, only 10 minutes marking my students' assignments, I am going to be busy for two and a half months, from eight to five, every day.”* (P15, female, lecturer, Faculty of Economic and Management Sciences)

“*I was literally starting to work four days a week at six o'clock in the morning, and then working a full day. And then, coming home and having home responsibilities, was also quite - it was like a dual thing. I was finding I was on the verge of burnout*.” (P17, female, senior lecturer, Faculty of Dentistry)

P20 has summarized this, as

“*[W]ork is hectic”*(P20, female, lecturer, Faculty of Arts and Humanities)

As far back as 2008, Barkhuizen and Rothmann indicated that due to a surge in work demands, academics are forced to work long hours. This puts them at risk for physiological, psychological, and behavioral illnesses. For example, Bezuidenhout (2015) found that South African academics' typical work week comprises of being a subject expert, researcher, lifelong learner, tutor, organizer, therapist, and appraiser. Furthermore, South African academics have substantial workload and administrative burdens. This includes governance demands devoid of organizational and managerial support, job uncertainty, and poor remuneration, along with role ambiguity (Poalses and Bezuidenhout, 2018; Du Plessis, 2020).

#### Competing Time Demands

The third highest ranked job demands was aspects that related to *competing time demands*, thus having to deal with constant conflicting work priorities. Participants indicated the following:

“*There is an expectation to have certain information on short notice*” (P2, female, lecturer, Faculty of Arts and Humanities)

“*Endless meetings are a challenge, because they disrupt workflow*” (P8, female, associate professor, Faculty of Economic and Management Sciences)

“*It doesn't matter what time of the year it is, you are always under lots of pressure to get things done*.” (P7, female, professor, Faculty of Arts and Humanities)

Another frustration is the institution's policies and procedures that academics need to follow in order to get something done. Participants explain:

“*The bureaucracy that you have to go through in order to, for example, go to a conference. And then sometimes you even go through this whole admin process and you get rejected*.” (P5, female, senior lecturer, Faculty of Economic and Management Sciences)

“*The sort of paperwork and bureaucracy involved is a real hindrance.”* (P22, female, lecturer, Faculty of Arts and Humanities).

This has led to P8 feeling very “*frustrated with the constant red tape*.” In support of this is Rice and Sorcinelli (2002) and Ylijoki (2013) who indicated that universities put an extraordinary amount of pressure on academics whilst having limited time (and financial) resources with which to complete a task.

Furthermore, the additional hours of work that academics tend to put in during their own time is often not reflected in typical workload models. Thus, there is a concern that academic environments will no longer be regarded as a better career option in terms of work-life balance (Dhanpat et al., 2019) but shall instead turn into an environment that is characterized by constant pressure if the movement toward ever increasing productivity (Kinman, 2014) does not level off (Callaghan, 2016).

### Qualitative Demands

The themes identified as qualitative demands focus on skills and/or effort required to complete work tasks. In this case it was not about the volume of the work, but rather the complexity of work tasks requiring cognitive, emotional, or physical skills and/or effort.

#### Balancing of Work and Home Responsibilities

*Balancing of work and home responsibilities* were also mentioned frequently, and ranked as fourth most taxing job demand. Participant 20 mentioned that “*work extends into family time*.” In addition, P7 indicated that in order to manage the workload, one has to “*work on weekends*” and this created a “*lack of work-life balance*.”

“*Remember, I'm a mother, I'm a wife, I'm a student, I'm a lecturer. I feel guilty that I'm not paying attention to my family as I am forever either in meetings or chasing deadlines.”* (P4, female, lecturer, Faculty of Economic and Management Sciences)

“*It needs to be noted somewhere that a workload isn't just your work from nine o'clock till five o'clock. It actually extends into your after hour time as well.”* (P11, female, lecturer, Faculty of Community and Health Sciences)

“*When you leave the office, your work is not done. Although we have flexibility, you find yourself working into the evening, weekends, Saturdays and Sundays.”* (P7, female, professor, Faculty of Arts and Humanities)

Although universities might offer comparatively flexible working hours when compared to other industries, there is a large probability that surmounting pressures on academics can lead to them working similar hours and even taking work “home” (Callaghan, 2016).

That is, participating in the work role makes participating in the family role more difficult and vice versa. It can thus be noted that where performance objectives, work hours and duties of employees become unrealistic and extreme—to the point that work incessantly places restrictions on academics' personal lives—they will experience an inability to relax (even after hours) due to the huge amount of pressure they face daily (Parker and Hyett, 2011).

#### Complexity of Student Support

Diversity amongst students was something that many academics had to deal with. Diversity components mentioned included aspects such as race, sex, culture, religion, age, and language. Although the medium of instruction of the research university is English, most students do not have English as their mother tongue. The diverse student base is a direct result of Higher Education initiatives to ensure equity of access to students. Participants share their experiences:

“*The challenging aspect in handling a very large class is that the students are diverse. So it becomes more challenging to communicate*.” (P10, male, senior lecturer, Faculty of Natural Sciences).

“*Language for me is also a challenge.”* (P1, female, lecturer, Faculty of Community and Health Sciences).

“*They* [referring to students] *feel themselves that they are being discriminated against. It is always difficult to gain their trust. I talk to them about developing as South Africans and finding ways to get them to understand one another.”* (P2, female, lecturer, Faculty of Arts and Humanities).

The majority of the participants felt that they put a lot of effort into their teaching and learning strategy (P1, P2, P4, P13, P18, and P23). Frustrations experienced by participants include the following:

“*The students will be demanding, harassing actually, in their e-mails demanding for examination scope.”* (P16, male, senior lecturer, Faculty of Law).

“*What makes it difficult is the social issues, the economic struggles of our students. They miss class because some don't have transport money to come to varsity.”* (P2, female, lecturer, Faculty of Arts and Humanities).

While students are encouraged to be responsible for their own learning (Lavhelani et al., 2020), it still remains the responsibility of the academic to ensure a high success rate when it comes to student learning. This causes an undue amount of stress for the academic as the level of student success determines how successful academics are within their roles (Dey et al., 2015).

#### Organizational Politics

*Organizational politics* seemed to have impacted most of the academics interviewed. Whilst the volume and time needed to complete the bureaucratic processes featured as a quantitative demand, it became clear that participant also experienced complexity and emotional effort as a result. For instance, there was constant mention of competition amongst staff members. Participants mentioned:

“*We have leaders that have an agenda. They are not always neutral*.” (P4, female, lecturer, Faculty of Economic and Management Sciences)

“*When you come into a setting, people make sure they are on the positive team end, meaning that the highest loads would be delegated to the newer novice and younger academics.”* (P14, male, lecturer, Faculty of Community and Health Sciences)

“*Since I am younger that the other academics, I don't have a voice. If I do speak up in a meeting, nothing gets done and it doesn't really get heard.”* (P15, female, lecturer, Faculty of Economic and Management Sciences).

Rice and Sorcinelli (2002, p. 104) have put forth similar evidence, whereby junior academics are “awkwardly clasped between local and cosmopolitan pressures, amongst disciplinary colleagues and organizational demands.”

Another element that came about was the lack of mentorship received for novice academics. This seemed to have a negative effect for participants, such as:

“*You are given a task, and you must find your own way.”* (P4, female, lecturer, Faculty of Economic and Management Sciences)

“*I learnt by knocking my head. Although my mentor was there, he was focussed on his own career.”* (P6, female, associate professor, Faculty of Natural Sciences)

“*My experience with my work was very challenging. You don't get mentored into your position. You just get told this is your job and you need to find your way.”* (P3, female, lecturer, Faculty of Community and Health Sciences)

Effective mentoring is vital and is needed to share imperative knowledge, abilities and insight with an employee that is new in the workplace. Paris (2013) puts forward that the novice lecturer is normally ready and willing to benefit from such an interchange so as to improve his/her professional journey. Lecturing is a very demanding and a stressful job, particularly for new lecturers. Therefore, new lecturers often have trouble transitioning into their new roles from learners to teachers or from industry to classroom (Franklin and Molina, 2012). Adizu and Effiong (2020) indicated in their study that the observed benefits of effective mentorship can lead to professional development of the mentee along with progression in pedagogical knowledge.

#### Lack of Mental Health Support for Academic Staff

The majority of participants indicated that there is a lack of consideration for academic staff's mental well-being (e.g., P3, P4, P6, P8, P13, and P17). This is in addition to the fact that students are put at the forefront with “*their needs being catered to first*” (P3 and 13). Participants explain:

“*There is no emotional support. Even if it's there, it's not genuine. Maybe I am crazy, but I am expecting a call from my superior asking me “hey, how are you? Are you coping?” You're not getting any of that.”* (P4, female, lecturer, Faculty of Economic and Management Sciences).

“*Sometimes we forget about the staff component, we are doing everything for the student. They need to think about staff wellness and mental health of staff as well.”* (P3, female, lecturer, Faculty of Community and Health Sciences)

P11 mentioned that academics have to deal with students' mental/personal problems, which they themselves are not equipped to deal with. This has caused a lot of anxiety, as there is “*limited counseling for the staff members*” dealing with these types of issues.

Thus, it is imperative that HEI's ensure that the mental well-being of staff is prioritized as they rely on the dedicated efforts of all staff members to allow for a successful workplace. Nevertheless, if occupational demands overshadow occupational resources, work tends to be more challenging and stressful, which is preceded by an exhausted, disengaged workforce (Poalses and Bezuidenhout, 2018).

### Organizational Demands

Organizational demands are described as those elements that are brought about by aspects or changes in the work environment. These aspects may have a negative impact an individual in terms of their work outputs (Bakker et al., 2004).

#### Using Technology-Mediated Learning Approaches

A number of academics indicated difficulty in dealing with technology. Especially as it pertains to emergency remote learning due to the COVID-19 pandemic. Academics had to learn to navigate their way “technologically” as this became a requirement.

“*Technology is a challenge for me. Even iKamva [referring to the online learning management system].”* (P9, female, lecturer, Faculty of Dentistry)

“*For me, the biggest challenge is this sort of e-learning, and the use of technology and the use of different modes. I always need to phone people and say ‘can you help me'. That's like a headache for me.”* (P11, female, lecturer, Faculty of Community and Health Sciences)

“*I like to work on the hard copies and I'm not keen to do everything electronically*.” (P2, female, lecturer, Faculty of Arts and Humanities)

The coronavirus SARS-CoV-2 has affected economic and social sectors across the world, including higher education in South Africa. Due to social distancing, various higher education institutions had to ensure that all learning material be placed online in order for students to access it. It has become essential for universities to not only offer theoretical learning but also to provide student with practical training by using technology. Additionally, institutions had to find alternative forms of formative (and most likely summative) ways in which to assess students. Most academic staff at contact universities normally' have limited, if any, knowledge or training in the instruction or delivery of online learning. As a result, academics with teaching responsibilities needed to develop their skills and become familiar with online learning platforms as soon as possible. This also includes a significant increase in administration (Hedding et al., 2020).

#### Lack of Structural Resources

Furthermore, it was highlighted by participants that certain *resources was required from the work environment* in order for them to be successful in their job roles. This was not always provided by the institution and thus participants indicated using their own resources or “just having to work with what they have.” For example:

“*I have to share my office with a colleague.”* (P1, female, lecturer, Faculty of Community and Health Sciences)

“*I use my personal laptop, rather than my computer at work, because I use specific programs for my course, the PC at work doesn't support this program. And also for say, taking photographs in the archives. I use my own resources.”* (P5, female, senior lecturer, Faculty of Economic and Management Sciences)

A healthy environment as discussed by Nordic (2009) in a faculty can be influenced by many factors. Whilst some of these factors are intrinsic, extrinsic factors, including the availability of university resources (Stankovska et al., 2017) were noted as important drivers of a healthy environment.

These types of resources mentioned date as far back as (1989) where Hobföll specifically mentioned that object resources play an important role in the stress experience of individuals (e.g., office space). It is thus evident that an absence of sufficient structural resources to perform the job effectively will cause an increase in the amount of pressure the individual will experience as they are trying to carry out their job tasks in the best possible way (Poalses and Bezuidenhout, 2018).

### Organizational Resources

Organizational resources is something that the institution would provide or the academic could use in order to alleviate the demand.

#### Social Resources

Having *social resources* where you know that you are not alone, was a key variable for most academics. Respondents spoke about having support from various sources. This included peer/social support, support from colleagues and supervisors and administrative support. This was summarized as follows:

“*I think because we're such a small department, we're quite a close-knit group”* (P5, female, senior lecturer, Faculty of Community and Health Sciences).

“*Okay, yes, generally it has been wonderful. Amazing actually. So my department supported me immensely”* (P16, male, senior lecturer, Faculty of Law)

“*There are offices that one can go to now and get advice, like teaching and learning. You can consult and get help with how to structure your course, and how to develop a new curriculum, and how to use the online resources in the teaching. That helps a lot, because you know that you're not alone*” (P6, female, associate professor, Faculty of Natural Sciences).

“*I think also with the opportunities that are provided by the different DVCs* [deputy vice chancellors]. *For example, the DVC of research when it comes to workshops and writing retreats, the Division of Postgraduate studies, organising all different workshops that are related to conducting research and otherwise, and also the availability of funding to apply for different projects*” (P12, male, lecturer, Faculty of Community and Health sciences)

Managers and supervisors should assist in instilling and upholding an encouraging workplace environment and practices as this influences an employee's job demands and resources (Van den Broeck et al., 2008; Alzyoud et al., 2015; Kotze, 2018). Theron et al. (2014) conducted a study which revealed that academic staff received adequate support from their managers but felt that more emphasis needed to be placed on aspects such as performance management and feedback needed for improvement (Lesenyeho et al., 2018).

### Personal Resources

Personal resources were identified as state-like aspects of self that, in part, determine and influence individual's abilities to impact of control their environments (Xanthopoulou et al., 2009). Therefore, it is seen as personal or psychological resources orienting from the individual.

#### Autonomy

Most participants felt that they had a good measure of control when it came to managing their work. Decision-making freedom and autonomy was experiences were shared throughout interviews. Participants share:

“*Surprisingly, I have been given quite a good level of control. I was allowed to pick my subjects, as in what I wanted to teach.”* (P16, male, senior lecturer, Faculty of Law)

“*There is a lot of freedom to change things, or to bring about new ideas.” (*P11, female, lecturer, Faculty of Community and Health sciences)

Academic freedom matters and it matters a great deal (Nongxa, 2020). It has been noted that the level of autonomy and influence that academics have over their work, signifies a key element which directly impacts the level of job satisfaction (Bentley et al., 2013). In addition, it moderates the harmful effect of other factors, such as high work demands (Fredman and Doughney, 2012). Thus, when autonomy is applied with the right sense of duty and accountability, it will in all likelihood result in academics being able to excel in all relevant areas (Kori, 2016).

Another aspect where participants experienced autonomy was in the flexible work arrangements provided by the institution.

P23 indicated that having “*some level of flexibility*” provided opportunity to take time off to focus on research projects, which was summarized by P18 as a “*good opportunity for growth*.” Others relate:

“*That helps because I live far from the Faculty so it's much easier for me just to do my research at home”* (P23, female, senior lecturer, Faculty of Law). “*I mean the fact that we can have almost flexible time is a very, very good attribute that I can attest to that we see over the years. Nobody is standing behind me and you have to clock in and clock out now”* (P18, male, lecturer, Faculty of Art and Humanities).

In previous studies, results have indicated that Flexible Working Arrangements (FWA's) prolong employment (Damman and Henkens, 2018), improve work functioning (Amick et al., 2017), and postpones retirement among older workers (Moen et al., 2017). Dropkin et al. (2016) also argues that FWA's provide more comfort (such as being able to work from home), autonomy (having less face-to-face managerial supervision), and control (being able to control one's work hours) and reduces the amount of stress experienced, increases job satisfaction, and improves work–life balance (Vanajan et al., 2019). Furthermore, Bayissa and Zewdie (2010) indicated that prospective growth and career development opportunities, such as being able to further ones education, having job security and job autonomy, are some of the major rewards available to academic staff in higher education institutions.

#### Meaningful Work

Participants mentioned that they found purpose and meaning in their work. Even though a vast majority indicated that they felt overburdened by their work load, they also indicated that they experienced their jobs as meaningful. Comments such as “*the role brings a level of satisfaction*,” “*wanting to give back to society*,” this being “*their purpose*,” finds the “*role rewarding*” were all phrases used throughout. It can thus be assumed that participants find a deep sense of meaning in the work they do. In more detail, participants shared:

“*I get, in proportion to the frustrations of working at this institution, I get as much satisfaction from engagement with the students and being a part of their lives”* (P22, female, lecturer, Faculty of Arts and Humanities)

“*My motivation is giving back to society”* (P10, male, snr lecturer, Faculty of Natural Sciences)

“*Definitely making a difference. That's it, making a difference, and believing that this is what I was born to do”* (P20, female, lecturer, Faculty of Art and Humanities)

“*I love working with the students, and when you actually see that almost light bulb moment where the students say oh, I get it. This is what you have been speaking about. That is a very rewarding feeling*” (P13, female, lecturer, Faculty of Dentistry)

“*And to make a difference because I have that at my disposal”* (P21, female, lecturer, Faculty of Natural Sciences)

Even though HEI's are environments currently characterized by high levels of stress and pressure, research suggests that academic work is still seen, in most countries, as a job that brings about high levels of fulfillment (Bentley et al., 2013; Shin and Jung, 2014). This could be due to a number of reasons, including HEI's being workplaces that have positive social characteristics. This can be seen as critical determinants of an academic's level of job satisfaction. This includes aspects such as university atmosphere, sense of community, relationships with colleagues (Lacy and Sheehan, 1997), perceived quality of students (Bentley et al., 2013), effectiveness of administration and technical/administrative support (Rosser, 2004; Bentley et al., 2013), quality of academic leadership (Fredman and Doughney, 2012), or social reputation of academics in society (Shin and Jung, 2014).

#### Personal Support

*Personal support* was the final resource highlighted in the study. Chen and Fellenz (2020) found that individual's personal resources at home increased their personal resources at work. For example, participants indicated receiving a good level of support from their spouses. “*And my husband is also, I don't know if he counts as a resource but having his support helps me a lot*” (P23, female, senior lecturer, Faculty of Law)

This assisted in dealing with work stress as mentioned by P4.

“*How I cope, I don't know. It's by the grace of God, and also maybe my husband is also helping in that way”* (P4, female, lecturer, Economic and Management Science Faculty)

There was also having that “*understanding from family members*” when having to bring work home in addition to having a productive space and enough resources (such as “*internet, printer and a pc*”) when working from home.

“*Yes, for instance maybe if you are in the workspace, they give you that time to do your work. That is another way of support”* (P10, male, senior lecturer, Faculty of Natural Sciences)

“*So I have resources at home that I need in terms of access to internet, computer, space at home to work*” (P17, female, senior lecturer, Faculty of Dentistry)

According to Sonnentag (2017), beneficial work environments in the form of job resources as well as beneficial personal resources is needed in order for work engagement to transpire. In addition, Bakker and Demerouti (2007) and Herbert (2011) have indicated that how the employee perceives and uses their personal resources, such as resilience and optimism and positive self-evaluation, will determine how they control their environment.

## Discussion

The aim of the research was to explore how academics make sense of the job demands and resources they encounter in the Higher Education environment. The JD-R model was used as theoretical framework to guide the exploration of what demands and resources exist in the academic environment.

From the results, it can be noted that elements such as having a heavy workload, work pressure and constant research demands resulted in academics feeling anxious and tired. Constantly having to work in such a demanding environment results in low levels of teacher-efficacy, low levels of work engagement and burnout (Han et al., 2020). Furthermore, having a lack of teaching resources impacts academics' work-life balance as most academics indicated that it became difficult to separate home and work responsibilities. Thus, institutions need to ensure that the retention and career fulfillment of academic staff (Ng'ethe et al., 2012), along with academic well-being, be made a priority (Janse van Rensburg et al., 2018).

As Leibowitz et al. (2017) show, teaching in a poorly resourced context with a large staff-student ratio of largely under-prepared undergraduate students creates different demands on academic staff members. As evident in the theme *complexity of student support*, academics in this South African university not only needed to focus on their teaching, but also on the socio-economic and psychological needs of students. Hence the need for academic development support programmes at universities is highlighted. However, the level of support and other resources have declined (Barkhuizen et al., 2014a). As such, academics draw on their personal resources, such as meaningful work and making a difference in the lives of students, to buffer the effect of the demands. The majority of the respondents found great meaning in their roles as academics. Moreover, their main reason for staying in academia was because they knew they were able to make a difference for students as well in a broader context i.e., communities.

Out of the 22 participants that was interviewed, the majority of the participants indicated that the demand experienced as most challenging is *publication pressure*. Second to this, participants indicated that being *overburdened with the load* was quite demanding. C*ompeting time demands*, as third highest ranked job demand, spoke directly to the amount of work pressure that was being felt by academics. Participants mentioned that a lot of their time was being spent on lectures/teaching, countless meetings in addition to consulting with or assisting students. Such high workloads and large intake of students are likely to lead to a decline in commitment and job insecurity (Theron and Dodd, 2011), as well as low levels of well-being. The fourth most prominent demand mentioned was *work-family life conflict*. This could have been a direct result of the pandemic COVID-19 lockdown and the move to remote working with resultant utilization of online and technology-mediated teaching technologies.

Although there are still many challenges to overcome, there were also positive elements identified from the interviews conducted. Aspects such as autonomy, flexibility in ones work schedule and having the support of supervisors, colleagues, and peers appeared to make quite a difference.

The JD-R model proposes that the interaction between job demands and job resources is important for developing motivational processes leading to work engagement and well-being, as well as depletion processes of job strain that lead to burnout (Bakker et al., 2014). As we did not ask participants about the interaction of their job demands and resources, it is not clear how they made sense of this interaction. However, job resources particularly influences engagement and well-being when job demands, such as publication pressure and work overload are high. In line with the Conservation of Resources theory by Hobfall (1989), resource gain becomes more salient in contexts of resource loss. As one example gleaned from the across the interviews attest, publication pressure was buffered by participants' acknowledgment that they have complete autonomy in deciding what they want to research. Our interpretation of the interaction of job demands and resources of academics are purely speculative, however, there is reason to believe that personal resources of those who choose to be academics could be used in creative ways to buffer inescapable work demands.

### Limitations and Recommendations

The present study was not without limitations. It is important to emphasize that the findings of this study were related to academic employees in a Higher Education Institution in South Africa, and it cannot be assumed that the results would be applicable to all other settings and other Higher Education Institutions. Another possible limitation is that participants may have felt hesitant to share certain information considering the sensitive nature of the topic being discussed. However, the interviewer felt contented that an acceptable level of care was in place to ensure that open and honest feedback was shared by participants.

For future research, it is recommended that a longitudinal study exploring job demands and job resources from the perspective of academics be identified over a period of time. This could add to a better understanding of changes taking place within the Higher Education Sector. It is also suggested that future research concentrates on discovering ways in which higher education institutions can provide support to employees to assist them in better dealing with the demands experienced, and provide employees with training on how to better manage their resources to enable them in dealing with challenges faced in work and life. This could include training to empower employees with skills to effectively raise concerns around demands being experienced in the workplace, and help employees to explore alternative ways of achieving the desired results through open and candid dialogue or job crafting interventions (Van Wingerden, 2016). A further recommendation is to explore areas of positive deviance. This could involve investigating areas in other sectors where there is a lower incidence of employees being at risk of burnout and exploring the factors present in the environment that could contribute toward this outcome, for example, increased work engagement (Alzyoud, 2016; Gauche et al., 2017).

## Conclusion

The present study provided insight into how academic employees make sense of the demands and resources in their job roles. Mention was made of how demands cause strain and stress, whereas resources was linked to the meaningfulness of the job. Research has progressively demonstrated that not considering the proper management of job demands and resources of one's workforce as a key element, can result in negative setbacks (Dhanpat et al., 2019).

By understanding the lived experiences of academics, leaders and human resource departments in HEI's could consider suitable interventions to alleviate job demands and implementing methods in which to structure work in order to increase resources at academics' disposal (Van Wingerden, 2016; Bakker and Demerouti, 2018).

Higher Education Institutions and key stakeholders should therefore be encouraged to reflect on the findings of the current study to ensure that the most appropriate method in which to offer ongoing support to their employees is considered.

## Data Availability Statement

The raw data supporting the conclusions of this article will be made available by the authors, without undue reservation.

## Ethics Statement

The studies involving human participants were reviewed and approved by Human and Social Science Research Ethics Committee of the University of the Western Cape. The patients/participants provided their written informed consent to participate in this study.

## Author Contributions

All authors listed have made a substantial, direct and intellectual contribution to the work, and approved it for publication.

## Conflict of Interest

The authors declare that the research was conducted in the absence of any commercial or financial relationships that could be construed as a potential conflict of interest.
